# Clustering of atomic displacement parameters in bovine trypsin reveals a distributed lattice of atoms with shared chemical properties

**DOI:** 10.1038/s41598-019-55777-5

**Published:** 2019-12-17

**Authors:** Viktor Ahlberg Gagnér, Ida Lundholm, Maria-Jose Garcia-Bonete, Helena Rodilla, Ran Friedman, Vitali Zhaunerchyk, Gleb Bourenkov, Thomas Schneider, Jan Stake, Gergely Katona

**Affiliations:** 1 Department of Chemistry and Molecular Biology, University of Gothenburg, Gothenburg, Sweden; 2 Department of Microtechnology and Nanoscience, Chalmers University of Technology, Gothenburg, Sweden; 3 Department of Chemistry and Biomedical Sciences, Linnaeus University, Kalmar, Sweden; 4 Department of Physics, University of Gothenburg, Gothenburg, Sweden; 5 European Molecular Biology Laboratory Hamburg Outstation, EMBL c/o DESY, Notkestrasse 85, 22603 Hamburg, Germany

**Keywords:** Biological physics, Condensed-matter physics, Structure of solids and liquids, Structural biology, X-ray crystallography

## Abstract

Low-frequency vibrations are crucial for protein structure and function, but only a few experimental techniques can shine light on them. The main challenge when addressing protein dynamics in the terahertz domain is the ubiquitous water that exhibit strong absorption. In this paper, we observe the protein atoms directly using X-ray crystallography in bovine trypsin at 100 K while irradiating the crystals with 0.5 THz radiation alternating on and off states. We observed that the anisotropy of atomic displacements increased upon terahertz irradiation. Atomic displacement similarities developed between chemically related atoms and between atoms of the catalytic machinery. This pattern likely arises from delocalized polar vibrational modes rather than delocalized elastic deformations or rigid-body displacements. The displacement correlation between these atoms were detected by a hierarchical clustering method, which can assist the analysis of other ultra-high resolution crystal structures. These experimental and analytical tools provide a detailed description of protein dynamics to complement the structural information from static diffraction experiments.

## Introduction

Trypsin-like serine proteases are activated by heat, which point out that vibrations play a crucial role in their function. Trypsins catalyze the cleavage of peptide bonds in the gut and their catalytic activity increases with temperature (up to an optimal temperature)^1^. Trypsin is a globular protein and its folded structure is maintained in a wide range of aqueous solutions. This structure forms spontaneously below a melting temperature, but the conversion from the inactive trypsinogen to the active trypsin form requires the cleavage of the Lys-15/Ile-16 peptide bond by an enteropeptidase or an already active trypsin. Premature activation of trypsinogen in the acinar cells or the pancreatic duct leads to tissue damage, which contributes to the development of pancreatitis. The conversion between the active and inactive forms involves a conformational change, which is thermally activated^2^. Denaturation of serine proteases change their low frequency vibrations^3^. In addition, vibrations were suggested to influence the catalytic activity of trypsin^4,5^. Under certain conditions, the low-frequency vibrational modes can be described by a canonical (Boltzmann) probability distribution. Such a distribution is necessary to model enzyme reaction kinetics^6,7^. The ideal temperature dependence of catalytic rates is usually only observed in a narrow temperature range in enzyme catalyzed reactions^8^. Fröhlich proposed an alternative to the canonical probability distribution (commonly referred to as Fröhlich condensation)^9^, which may be more appropriate for non-isolated biological dissipative systems. In this out-of-equilibrium model, the probability distribution is skewed towards the lowest frequency mode in a coupled oscillator system. Different degrees of condensation have been investigated using simulations, which revealed that the rates of excitation, the exchange between the vibrational modes, thermalization and the temperature of the environment are important factors^10–14^. Collective modes in biological systems are postulated to play a role in long-range protein-protein interactions, the adjustment of biochemical reaction rates and information processing in semicrystalline, supramolecular systems^10,15^.

Dissipation can facilitate disorder to order transition in different types of condensed matter systems. External terahertz fields were shown to lead to ordering in amorphous sorbitol even at cryogenic temperatures^16^. In solution phase (poly(3-hydroxybutylate) (PHB)/chloroform mixtures) microcrystal formation was also observed following pulsed terahertz radiation^17^. Recently, structural changes were observed in lysozyme crystals when pumped with 0.4 THz radiation at room temperature^18^. In agreement with these findings, continuous excitation of fluorophore-labelled bovine serum albumin with visible light has resulted in a terahertz spectral signal, which evolved over minutes time scales^19^.

In NMR experiments using the serine protease inhibitor eglin c as model protein, dynamics of amino acid residues were affected by distant, relatively conservative mutations^20,21^. Site-specific mutations also affected functional motions far away from the mutational site in a kinase recognition loop^22^. Furthermore, it is frequently observed that a mutation or binding of a small molecule at one site can affect the dynamics at remote sites and computational studies could capture such aspects of correlated motions in proteins^23,24^. As a general trend, long-range allostery is modeled as a network of interactions from one point to another where the diffusive motions are transmitted stepwise through a chain of local interactions between the distant sites^25–27^. Of note, local interactions still dominate in these network models and hence the influence they exert is expected to decay with increasing distance as they propagate in space. Reactive, near-field interaction of atoms with an oscillating electric field is not assumed to be necessary for explaining allosteric changes. However, electric field pulses can cause reversible, concerted conformational changes at multiple locations in a protein in a similar manner as allosteric effect that connects distant sites together^28^. Whereas, slow, high-field electric pulses happen infrequently in biological systems near membranes, weak, evanescent terahertz fields are more common in aqueous solutions in the wake of thermal vibrations of partially or fully charged atoms.

The main methods to study low-frequency vibrations in proteins are terahertz spectroscopy, optical Kerr spectroscopy and inelastic/quasielastic neutron scattering. Terahertz spectroscopy^29^ was particularly successful in separating protein vibrations from those of the bulk solvent and was used to detect long-range vibrations in the protein scaffold^30,31^. Terahertz spectroscopic measurements also revealed long-range dynamics linked to the functional state of proteins^32–34^. Since the vibrational spectra lack resonant bands a few polynomials are sufficient to describe the entire terahertz region. Polarization varying anisotropic terahertz microscopy breaks this deadlock by recording two dimensional spectra from oriented proteins in crystals yielding dozens of absorption peaks^35^. Unfortunately, typical proteins contain thousands of atoms and many degrees of freedom. Consequently, the interpretation of the spectra critically depends on the assumed model of protein and solvent dynamics. In contrast, high-resolution X-ray crystallography recovers the three dimensional distribution of atomic displacements described with amplitudes and directions^36^. If the diffraction experiment is not time-resolved, the displacements are time averaged. Although the displacements are indistinguishable from a static distribution, since atoms move even at 100 K, they have a dynamic component. Nonetheless, the spatial distributions of every atom in the protein are observed, resulting in several thousand parameters. In this study, we demonstrate the use of high-resolution X-ray crystallography to study the terahertz dynamics of proteins. The dynamic component of the distribution was directly investigated by irradiating the crystals with terahertz radiation and compare spatial distributions to the dark state.

**Figure 1:**
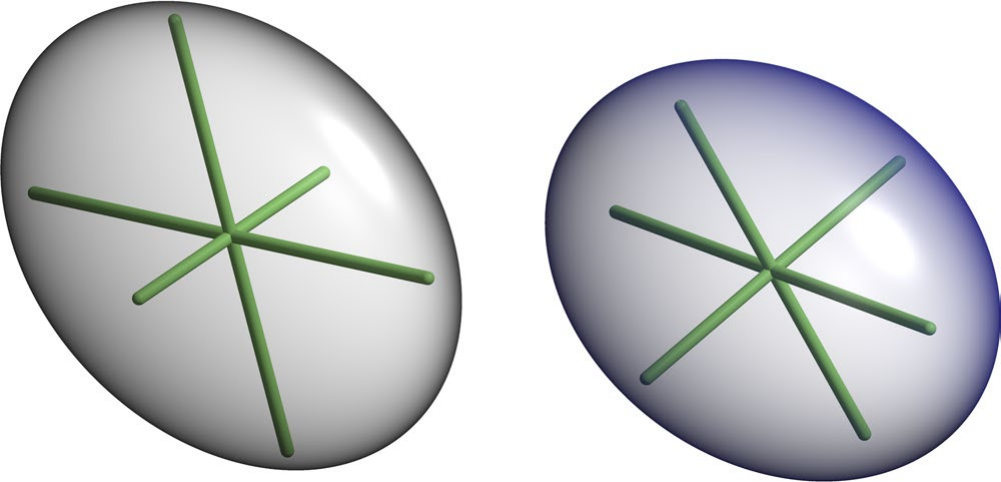
Thermal ellipsoids of the carbonyl carbon (left) and amide nitrogen (right) atoms of Thr-125B located close to each other. The carbonyl carbon and amide nitrogen atoms display slightly oblate (pancake shape) and prolate (cigar shape) ellipsoids, respectively. The direction and length of the axes in the ellipsoids are uniquely defined by eigenvectors and the corresponding eigenvalues of the ADP tensors. These two atoms are located in close vicinity to one other, but they display the highest degree of similarity with more distant atoms. The figure was obtained using RASTEP^36^, numbering of amino acid residues follows the chymotrypsin convention^66^.

The basis of our analysis involves the six atomic displacement parameter (ADP) tensor components, which can be illustrated by thermal ellipsoids after diagonalization (Fig. 1, Eq. 1). The axis orientations of the ellipsoids represent the eigenvectors of the ADPs and the amplitudes of the axes correspond to the eigenvalues. For a typical protein atom, the amplitudes are not equal (the atom is anisotropic). To simplify the comparison of the ADPs, we adapted the B_eq_ (which are analogous to isotropic B-factors, Eq. 2)^37^ and anisotropy metrics^36^ (henceforth ANISO, Eq. 3). ANISO varies between 0 and 1, where 1 indicates perfect isotropy.

The amplitude and direction of atomic motions are valuable constraints for dynamic models. However, the complexity of protein crystal structures makes it difficult to comprehend the patterns that may correspond to the different dynamical models. We solve this problem by applying an unsupervised machine learning technique: hierarchical clustering. The inclusion of all the information from the ADPs dramatically improved the specificity of the clustering. Groups of atoms that share similar dynamics could be inferred from the similarity of their positional probability distributions.

In this study, the non-thermal effect of 0.5 THz radiation was investigated in bovine trypsin crystals cryo-cooled to 100 K. We analyzed the changes in the ADPs upon terahertz irradiation in order to reveal the dynamical component in the displacements. We discuss the eigenvalue magnitudes of the ADPs on individual amino residue level by averaging the atomic data and we compare those results to simulated data. Finally, we elaborate on the effects on individual atoms related to terahertz dynamics using a clustering method, which analyses all elements of the ADP tensors.

## Results

We irradiated orthorhombic bovine trypsin crystals alternately with a 0.5 THz radiation, such that the odd frames correspond to “THz on”-states and the even frames correspond to “THz off”-states. As a reference, we performed the same data analysis on X-ray diffraction data collected from crystals that were not exposed to terahertz radiation. In contrast to an earlier experiment with hen egg white lysozyme^18^, bovine trypsin crystals were cryo-cooled to 100 K during the entire period of data collection with a dry nitrogen gas stream and suspended in a mylar loop without a surrounding plastic capillary. In total, five terahertz irradiated and four reference crystals were retained for analysis. Data processing statistics are summarized in Table S1.

### Comparison of the irradiated and non-irradiated states of the crystals reveal differences between individual residues

**Figure 2:**
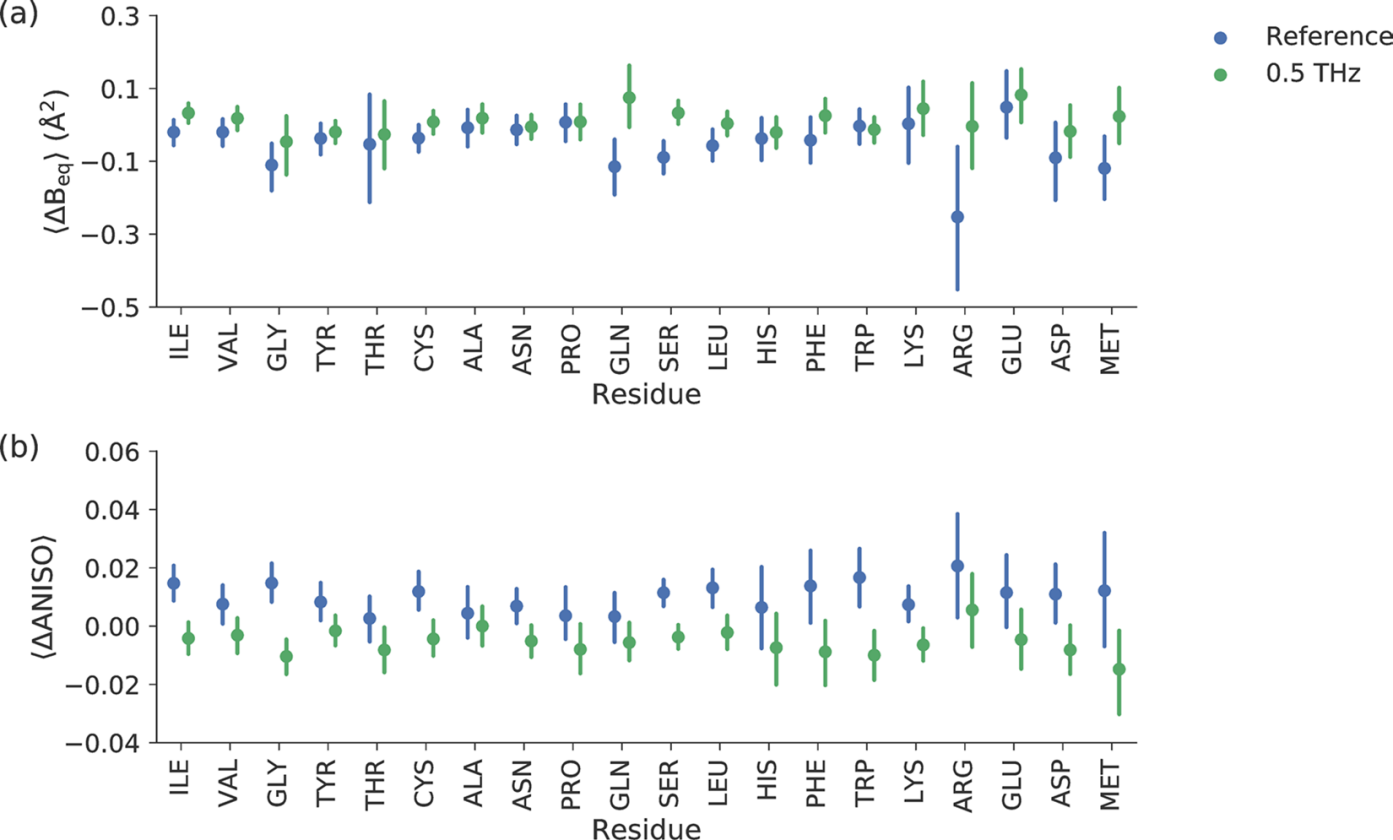
(**a**) B_eq_-factor change between crystal structures determined from odd and even diffraction images (B_eq,odd_-B_eq,even_). (**b**) The anisotropy value (ANISO) change between crystal structures determined from odd and even diffraction images (ANISO_odd_-ANISO_even_). Odd numbered diffraction images were recorded simultaneously with 0.5 THz irradiation in the THz irradiated crystals. *Blue* and *green* indicates reference (no terahertz irradiation at all) and terahertz irradiated crystals, respectively. Each average is calculated from the differences in B_eq_ and ANISO values from the atoms belonging to each amino acid types. The error bars represent 95% CI of the mean.

An initial comparison of the ADPs was based on the difference in B_eq_-factor between equivalent atoms determined from the odd and even frames (B_eq,odd_-B_eq,even_). The mean and confidence interval (95%) of the difference in B_eq_-factor for amino acid atoms were determined. Figure 2A shows this type of comparison for the twenty amino acids in the reference (*blue*) and terahertz irradiated crystals (*green*). B_eq_ appears to be either unchanged or similar to the references in the terahertz experiments except for the amino acid residue glutamine. The B_eq_ of glycine, glutamine, serine, leucine and methionine atoms appear to decrease in odd data sets in the reference experiments.

**Figure 3:**
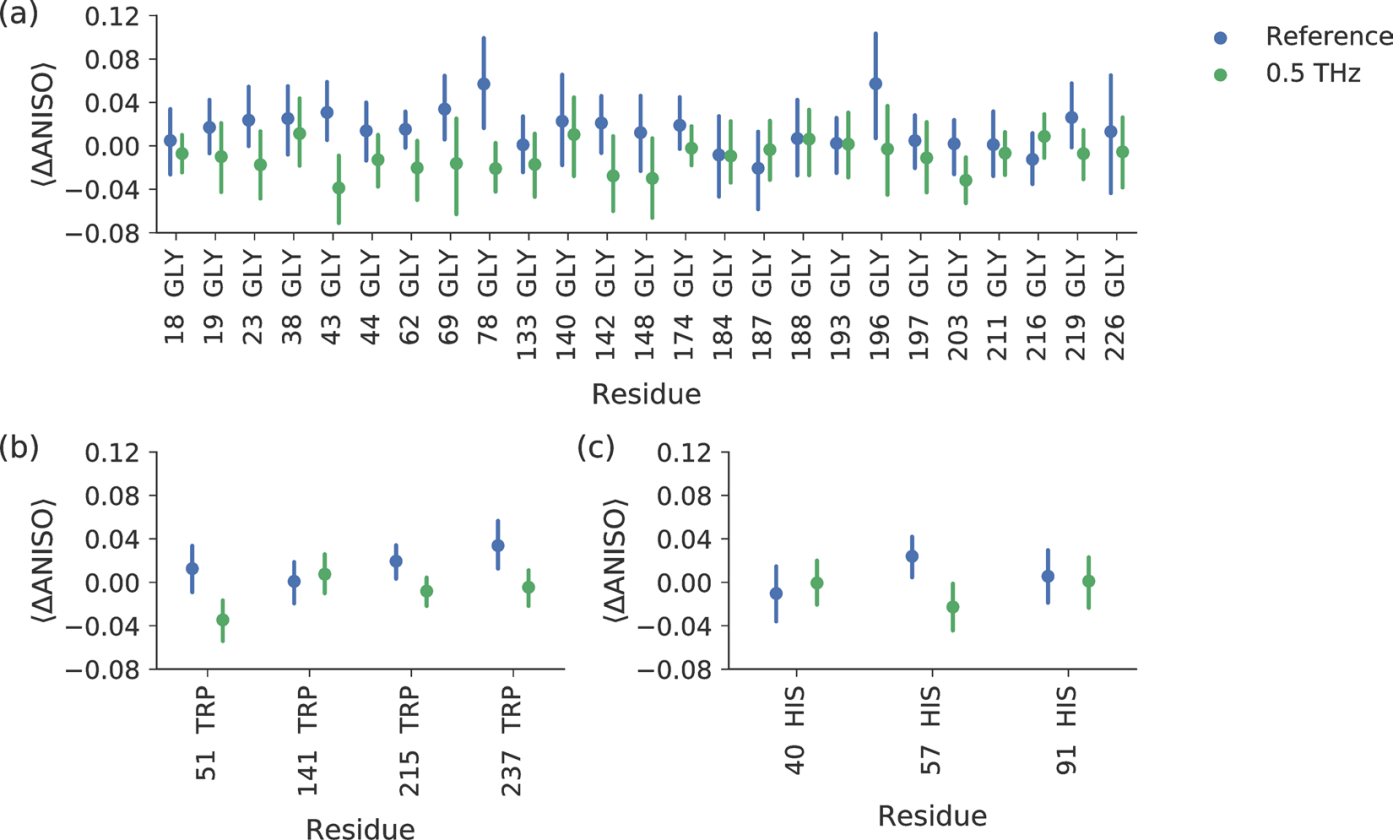
Comparison of ANISO change in individual (**a**) glycine (**b**) histidine and (**c**) tryptophan amino acid residues between odd and even diffraction images of the reference (*blue*) and terahertz irradiated (*green*) crystals. Each averaged difference is calculated from the differences in ANISO values. The error bars represent 95% CI of the mean.

The comparison of difference ANISO values (Fig. 2b) shows a limited overlap of confidence intervals (CI) between the reference and the terahertz categories. The greatest contrasts between the reference and terahertz categories are observed in atoms from isoleucine, glycine, cysteine, serine, leucine, tryptophan, lysine, asparagine and aspartate residues. In all these cases, the average ANISO of atoms decreased (i.e., they became more anisotropic) upon terahertz irradiation. The position of the amino acid residue in the structure is important, as shown by the ANISO value distribution of individual glycines, tryptophans and histidines are shown in Fig. 3. These amino acids do not have alternative conformations in the structure, which facilitates the comparison between different sites. From this comparison, it is obvious that most of the glycine residues behaved similarly in the terahertz radiation experiments compared to the references (Gly-43 and Gly-78 are exceptions), although in general, the atoms of the glycines in the N-terminal domain had a tendency to become more anisotropic when the crystals were subjected to terahertz radiation. Tryptophan residues became more anisotropic when all atoms were pooled together (Fig. 3) and Trp-51, Trp-215 and Trp-237 had different average anisotropies in relation to the reference, whereas Trp-141 stayed similar. Histidine residues, except catalytic His-57, appeared to be completely unaffected by terahertz radiation. As a general tendency, when there was a reversible change in anisotropy upon terahertz irradiation, it predominantly resulted in more anisotropic behavior in the residue atoms.

### Comparison to NMA and MD simulations

In order to model ADP tensor components (U_ij_) we performed NMA on an energy-minimized trypsin structure and generated random samples of the structure using all normal modes. The calculation assumed a temperature of 100 K. The B_eq_s of predicted atomic U_ij_s were approximately two orders of magnitude smaller than the experimentally observed values. Since NMA only explores the potential surface around a single minimum, it has a limited ability to follow protein dynamics, which consist of multiple conformations that exchange at slower than picoseconds timescales. Moreover, NMA is performed in a vacuum, which is not the case with solvated forms of proteins. Nevertheless, NMA is useful for inferring protein motions especially when such motions stem from the structure rather than the solvent dynamics, as is the case with cooled crystals, and hence we examined the correlation of NMA predicted U_ij_ metrics with Molecular Dynamics (MD) simulations and also with the experimental data.

**Figure 4:**
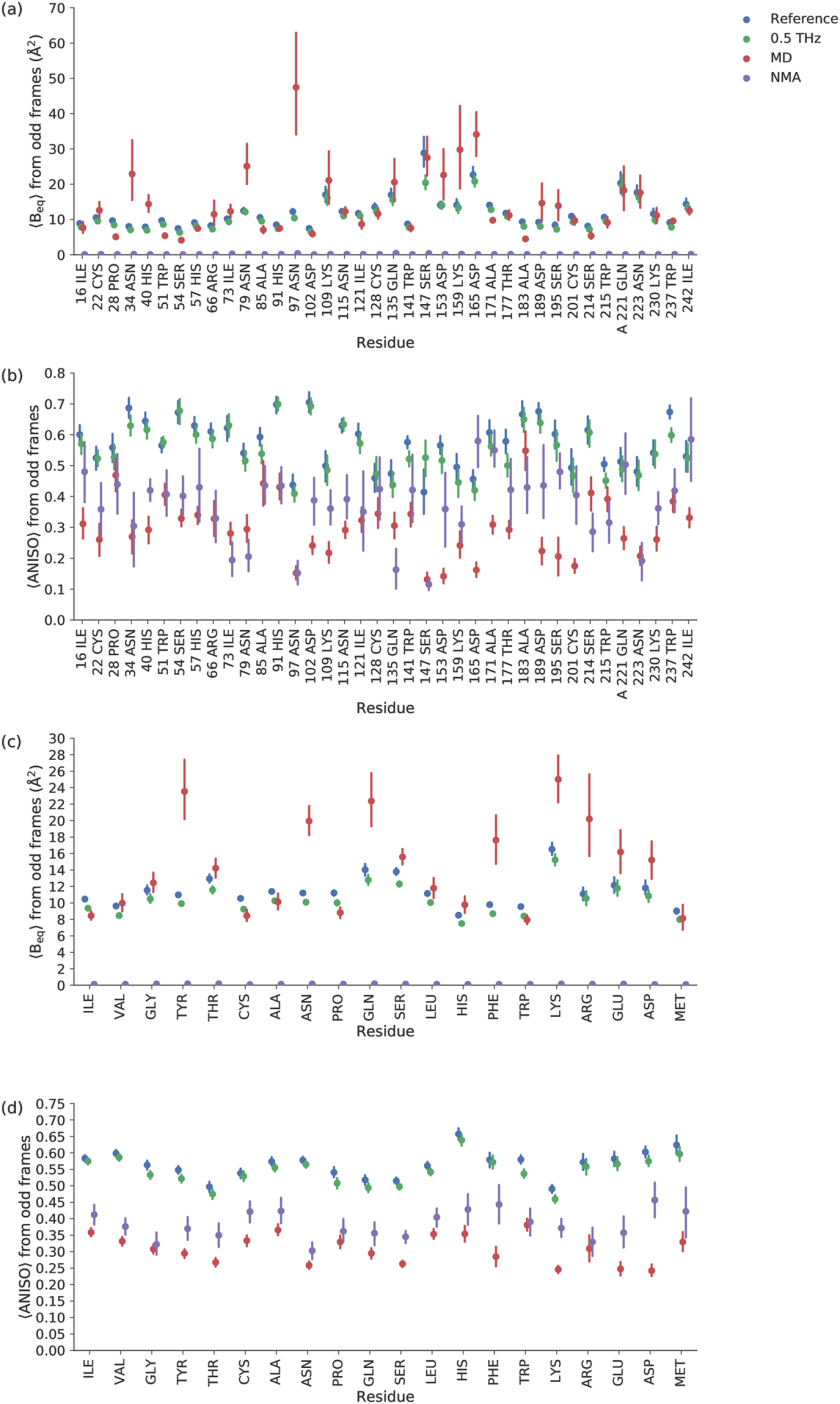
(**a**) Comparison of experimentally determined and simulated average B_eq_-factor in individual amino acid residues. (**b**) Comparison of average ANISO in individual amino acid residues. (**c**) Comparison of experimentally determined and simulated average B_eq_-factor in different amino acid types. (**d**) Comparison of average ANISO in different amino acid types. *Blue* and *green* correspond to the reference and terahertz irradiated crystals, respectively. *Orange* and *purple* indicate the predictions from molecular dynamics simulations and normal mode analysis, respectively.

An alternative to NMA is MD simulations of solvated protein molecules. Due to the fact that MD force fields are defined at 300 K, and to facilitate conformational changes, we performed the simulations at 300 K. When flash-cooling proteins, conformational variation is locked into a static distribution, but still affects the displacement parameters. Since conformational sampling is more delicate than sampling from an NMA ensemble, we used four MD simulations to predict the B_eq_ and anisotropy of the protein atoms and compared the values obtained to the corresponding parameters of the refined crystal structure (Fig. 4). The predicted B_eq_ values were approximately on the same scale as the experimental values. They appeared to be underestimated for the well-ordered parts of the structure, but overestimated for the flexible regions. In particular, the B_eq_ of Tyr, Asn, Gln, Phe and Lys residues were greatly overestimated compared to the experimental data. There was a 0.53 Pearson correlation between the calculated (from segments of four times 10 ns simulations) and the experimental means of B_eq_ per residue (calculated for all equivalent atoms from the reference crystals). The mean ANISO values in amino acid residues showed a smaller correlation (0.39), and MD simulations generally overestimated the anisotropy. By contrast, ANISO values derived from NMA simulations were more isotropic and agreed better with the experimental data. MD simulations predicted Asn residues to be much more anisotropic relative to other amino acid types as well (Fig. 4d).

Perhaps it is unrealistic to expect good agreement of U_ij_ derived values between experiments and predictions on an absolute scale. B_eq_ was found to be on approximately the same scale in MD simulations. Nevertheless, one expects that B_eq_ will be estimated self-consistently and reproducibly by MD simulations. Since Pearson correlation coefficients (CC) are not affected by the absolute magnitudes of compared values, they are also a suitable measure for comparing crystal structure and predicted values (self- and cross-correlations). As Table S2 shows, the MD B_eq_ estimations are not stable and their correlation with the estimated B_eq_ decays over time. It does not matter which reference time point is chosen. For example, the simulated trajectory from MD1 had a B_eq_ CC of 0.83, 0.71 and 0.64 when compared to MD2, 3 and 4, respectively. Choosing MD2 as a starting point also shows a decreasing CC trend. NMA B_eq_ shows a substantially lower correlation with any of the MD predictions (the best correlation is 0.45). Moreover, when compared to the B_eq_ of crystal structures, earlier trajectories show higher correlations than later ones irrespective of the crystal structure to which they are compared (Table S3). NMA B_eq_ correlations with crystals is on par with some of the MD predictions (the best correlation is 0.45 to crystal x28 and x30). Reference crystal structures had B_eq_ CC in the range of 0.99–0.82 among themselves due to experimental and modelling variation (Table S4).

Anisotropy values showed a smaller correlation within the experimental data and also between the theoretical predictions, although the predictions seem to become more stable over time (Tables S5, S6) than the CCs for B_eq_. Nevertheless, the combined experimental and modeling errors make predicted and experimental anisotropy less correlated (CC < 0.4 for MD and CC < 0.3 for NMA, Table S7). As such, we also have to consider the mismatch of the absolute level of anisotropy values between experiments and model predictions (Fig. 4b and 4d). We provide additional discussion about the NMA and MD modelling in the Supporting Online Material.

### Metric-free clustering of atoms based on pairs of U_ij_ matrices

Hierarchical clustering is an exploratory, unsupervised machine learning technique, which we use for identifying similar atoms (in a large pool of atoms) described by many features (elements of ADP tensors). Hierarchical clustering was used in the crystallographic analysis, for example by grouping isomorphous crystals together^38^ or detecting structural similarity^39^. In the context of analyzing ADPs, clustering algorithms have not been used before. Our atomic clustering method was based on similarities between the elements of ADP tensors, quantified by the Euclidean distance between the elements of the ADP tensors (Figure S1, Eq. 4). This way, atoms with similarly shaped ellipsoids, similar size and displacement directionality are grouped together. The heat map represents the magnitude of the tensor elements, providing a quick visual feedback about the similarity and the nature of the remaining differences. By changing the definition of distance, it is possible to emphasize different aspects of ADPs, but these alternative measures should converge to zero between identical ADPs. Different distance metrics capture small changes from ADP identity similarly. We focused our attention on the tips of the branches where the similarity between atoms is the highest and the type of distance is the least important. Another reason why we focused on the highest similarity between atoms is that they occur less likely by chance.

Completely ignoring certain aspects of ADPs for example considering only the mean displacement, related to the sum of the diagonal elements of ADP tensor (B_eq_) yields a very coarse clustering. However, slight differences in shape and orientation are enough to direct atoms to specific clusters. In Figure S1, the atoms from terahertz-radiated crystals are represented by green regions and these appear to cluster together. These clustered atoms tend to be located in the core regions of the protein structure.

**Figure 5:**
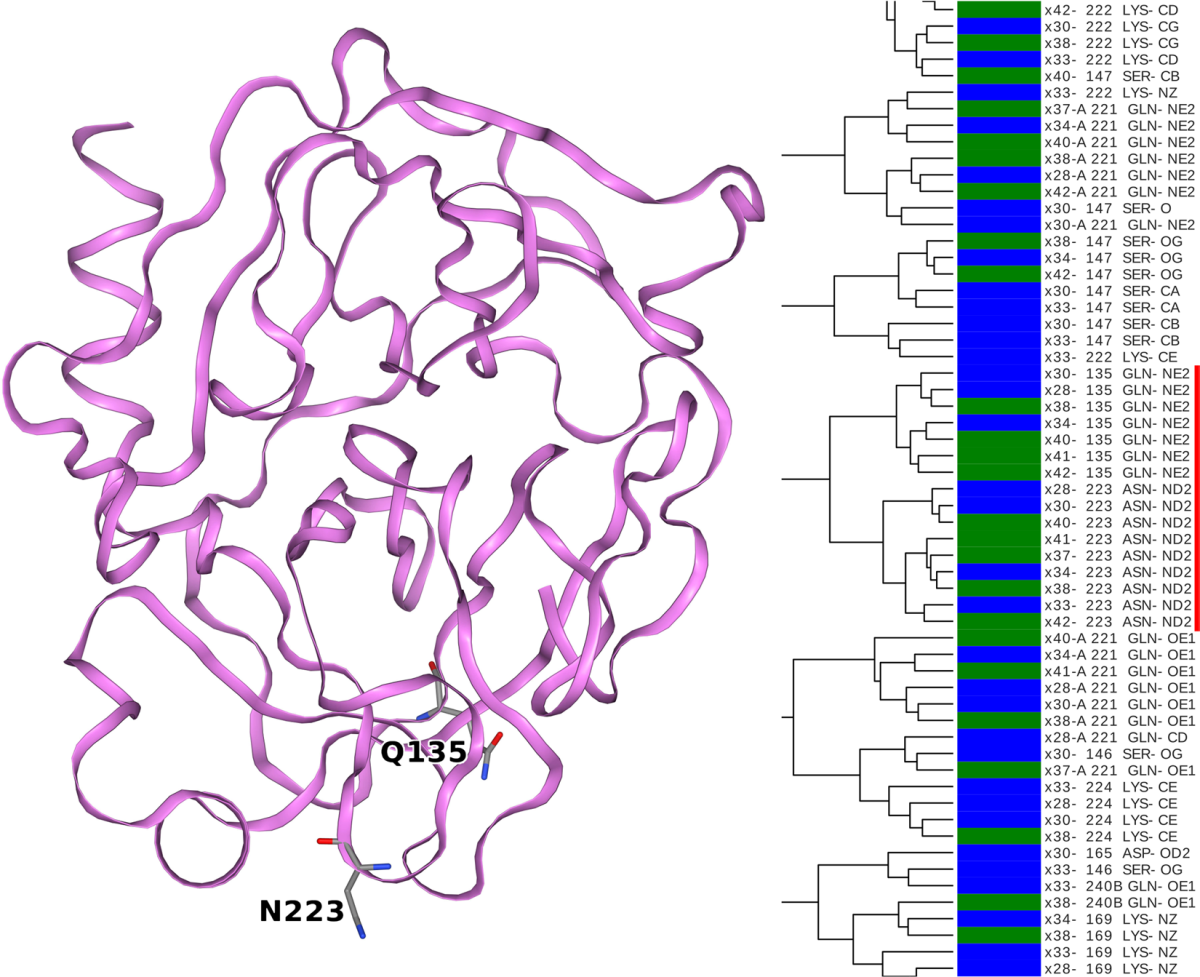
Clustering appears to identify amide nitrogen atoms of Gln-135 and Asn-223 residues in multiple crystals (*red*). The two amino acids do not make intra- or intermolecular contacts. The trypsin structure is illustrated by a ribbon diagram. Those amino acid residues to which the clustered atoms belong are represented by sticks.

**Figure 6:**
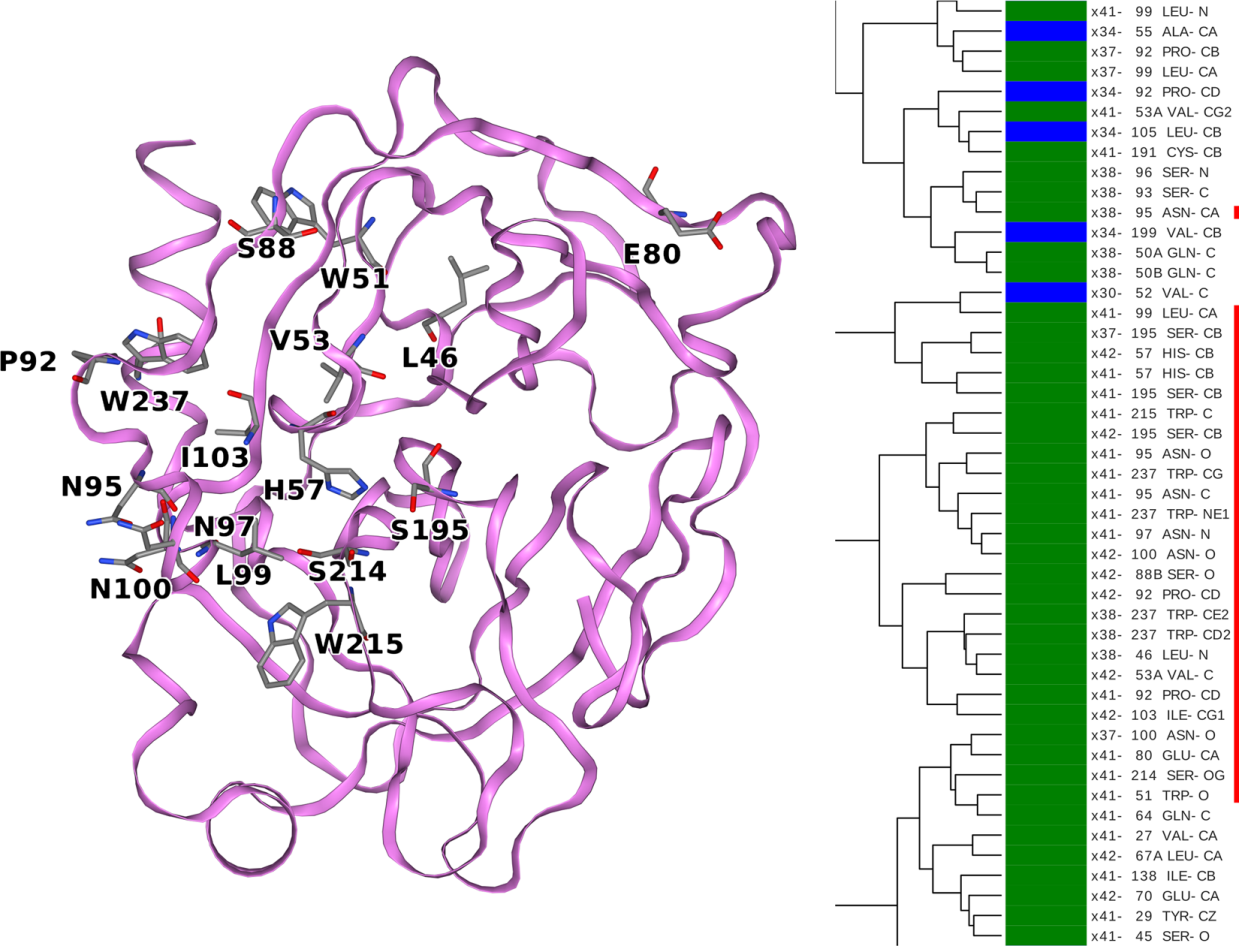
A cluster consisting of atoms from terahertz irradiated crystals that are involved in the catalytic process of the protein (Ser-195, His-57, Ser-214, Trp-215) and a high proportion of atoms originating from asparagine and tryptophan residues. The trypsin structure is illustrated by a ribbon diagram. Those amino acid residues to which the clustered atoms belong are represented by sticks.

**Figure 7:**
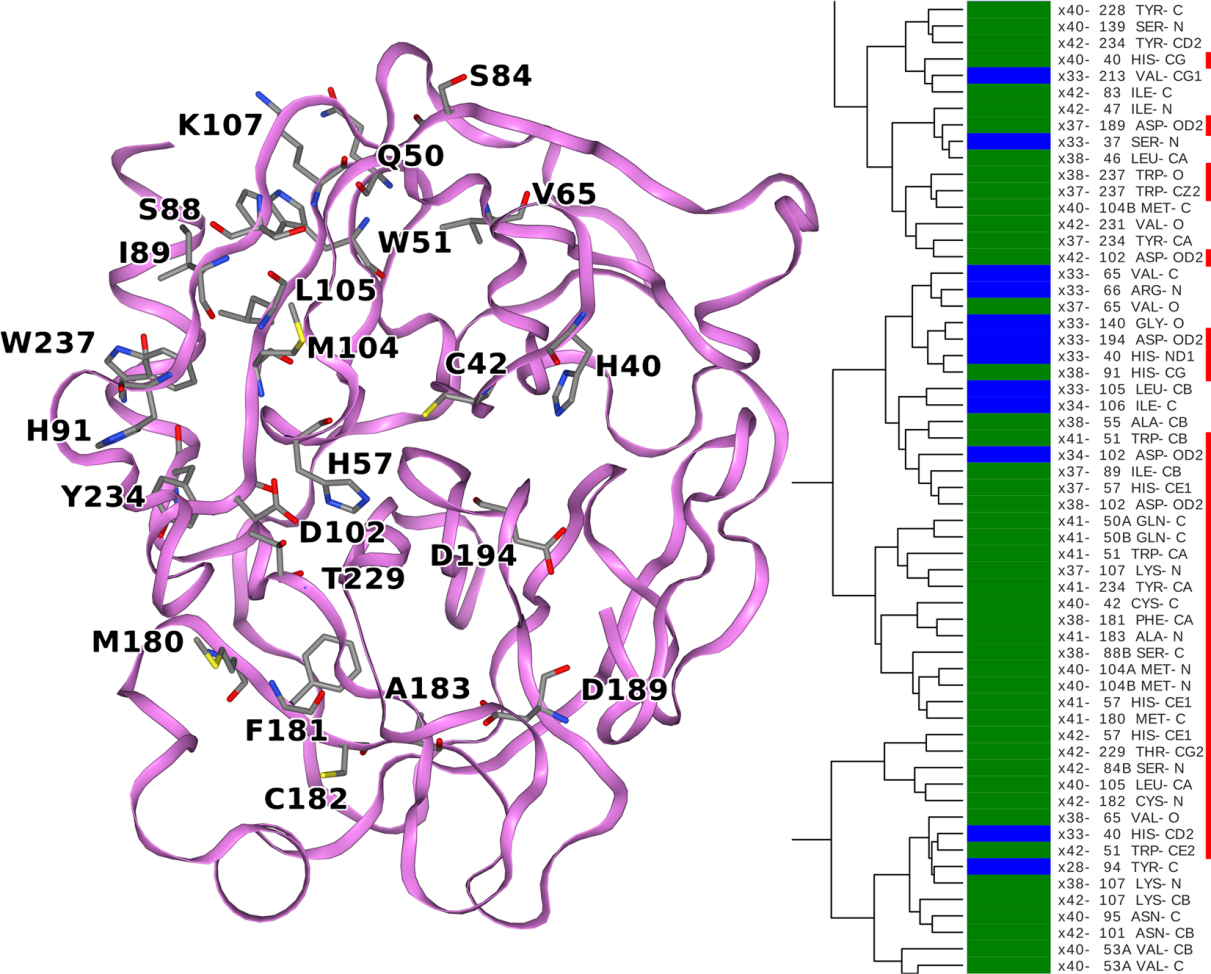
A cluster consisting of atoms from mostly terahertz irradiated crystals that are involved in the catalysis and activation of trypsin (His-57, Asp-102, Asp-194 and Asp-189).  A high proportion of atoms originates from tryptophan residues and all histidine residues are represented by atoms in the cluster. The trypsin structure is illustrated by a ribbon diagram. Those amino acid residues to which the clustered atoms belong are represented by sticks.

In order to illustrate different types of formed clusters, Figs. 5–7 highlights different regions of the cluster map, separated by thousands of atoms. Figure 5 zooms in on the top highlighted region in Fig. S1. Here, we examine 7 out of 9 observations of the N_ε_ atom of Gln-135 on one branch. The immediately adjacent branch on the dendrogram consists of the N_δ_ atom of Asn-223, where all of the observations of this atom in all crystals can be found. It is worth emphasizing that no other information was used other than the 2 × 6 elements of the U_ij_ matrices. Despite this, it is possible to uniquely identify up to 9 identical atoms from a total pool of 15921 atoms. Thus, ADPs are highly reproducible and weakly affected by experimental and modeling errors. It is important to note that these two atoms were not close to one other in the crystal structure, but they shared a similar position in a chemical group. The thermal ellipsoids these atoms are illustrated in Figure S2.

Figure 6 focuses on a central region highlighted in Fig. S1, where most of the clustered atoms share the fact that they were observed in terahertz pumping experiments. The highlighted cluster contains three observations of C_β_ of Ser-195 and two observations of C_β_ of His-57. Ser-195 carries out the nucleophilic attacks and His-57 accepts the protons generated during the nucleophilic attacks. The ADPs of the two C_β_ atoms are illustrated as thermal ellipsoids in Fig. S3. Atoms from Trp-215 and Ser-214 were also observed in this region. The backbone of these two amino acids form the antiparallel β-sheet with the substrate peptide in the Michaelis-complex and acyl-enzyme, and in addition the hydroxyl group of Ser-214 is hydrogen bonded to the third catalytic amino acid, Asp-102, which is not part of this cluster. The residue Trp-237 is represented with multiple atoms in the branch and this residue appears to be affected by terahertz radiation.

Figure 7 shows the lower marked region in Fig. S1, where the link between His-57 and Asp-102 appears to be stronger. In particular, there is a similarity between C_ε2_ of His-57 and O_δ2_ of Asp-102. In addition, other O_δ2_ atoms of functionally important aspartates seem to be spatially tuned together: Asp-194, which forms a salt bridge with N-terminal Ile-16 (a step necessary to convert the enzyme into its active form)^40^, and Asp-189, which (in general) forms the salt bridge with the P1 residue of the substrate. In this crystal structure, Asp-189 forms a salt bridge with the amidinium group of the inhibitor benzamidine. It is important to mention that atoms belonging to Trp-237 and Trp-51 also appear in these trees, as the anisotropy of these tryptophanes is affected by terahertz radiation.

## Discussion

### ADP similarity between chemically similar atoms

Our analysis revealed that atoms in functionally important residues appear repeatedly together in local clusters. Atoms in separate branches are often predominantly observed in either terahertz radiated or non-radiated crystals, respectively. In Fig. 6, one such terahertz radiated branch (green) is highlighted. The large physical distance between these atoms does not appear to be a hindrance, whereas the chemical nature of the atoms clearly does have an influence. For example, not all carbon atoms are preferentially associated with other carbon atoms, rather carbon atoms in carbonyl, α and β positions in the amino acid residues are different enough to sort them to different clusters. (Fig. S3–S5) The association between the chemical nature of atoms and their ADPs is an unexpected result and requires an explanation.

One could thus consider the ADP similarity to be an effect of the atom types having a similar partial charge. For example, such an assumption could be applied for the amide side chain nitrogen of asparagine and glutamine (Fig. 5), which are assumed to have a strong and similar negative partial charge in most empirical force fields. In a protein, there is a wide palette of atom types, and we approximate each as having a specific static partial charge between approximately −0.8 and + 0.9. Consequently, each of these atom types may have a slightly different response to oscillating EM fields. The partial charges are distributed alternately in the protein structure forming a mosaic of partial charges where positively charged atoms are usually bonded to negatively charged atoms. As a result, polar vibrations (optical phonons) could emerge when exposed to oscillating terahertz EM fields. Since the wavelength of terahertz (millimeter) EM waves is much longer than the size of protein molecules these fields are virtually uniform across the protein molecule at any given moment. Terahertz EM fields could originate from the thermal motions in the protein (crystal) and the solvent and these together generate evanescent fields. We also periodically applied an external terahertz field, but the combination of these field vectors also spatially uniform over a protein molecule and the surrounding solvent molecules. The association between the chemical nature of atoms and their ADPs is not fully explained by the arguments above. For example, C_β_ of Ser-195 is assumed partially positive, because of its bonding to the more electronegative hydroxyl group, whereas C_β_ of His-57 is assumed to be more neutral, because it is attached to another carbon of an imidazole ring. Nonetheless, these two C_β_ atoms have extremely similar ADP in several crystals (Fig. 6). If the two atoms of the same position (C_β_) are cluster together either they have a similar charge, which is not in agreement with the force field, but may be true due to local effects or it is not only the charge that is important.

### Do protein domain structures emerge from entrainment of atomic displacements

In architecture, one models the dynamics of an arbitrary structure (a house or a bridge) and then alters the structure until the dynamical properties are acceptable. Molecular NMA follows this same conventional logic, except that making planned changes in a protein structure is not a trivial process. In contrast to conventional engineering, a protein structure is not created by placing the atoms into arbitrary positions; rather, it emerges spontaneously from a quite dynamic polymer under suitable conditions (protein folding). ADPs of atoms do not (exclusively) depend on where they are located in the structure, which suggests that most atoms are not passive passengers in their structural elements.

We showed that in the folded, crystalline form of a protein, atomic fluctuations are characteristic, stationary (reproducibly seen in multiple crystal structures) and coordinated across long distances connecting together different structural regions. At first glance, intriguingly, atoms of a sharply defined chemical nature align themselves with similar atoms in other identical chemical groups. Another non-random aspect is that atoms cluster together when they originate from terahertz pumped crystals (Figs. 6, 7 and S4). Terahertz radiation induced ordering is not specific to proteins and aqueous solutions, but it has been observed in amorphous solids and non-aqueous solutions^16,17^. Trypsin crystals are well ordered even before terahertz irradiation, but the dissipation of applied terahertz radiation enhances the clustering of atoms. Atoms from terahertz irradiated crystals more likely cluster together. Terahertz irradiation also increases the anisotropy of atoms in a large fraction of tryptophan residues. On the other hand, the ADPs in our reference crystals already provide substantial information about the long-range pattern: the pattern is not exclusively present in an over pumped condition.

In a protein crystal structure some, but not all, chemically similar atoms have similar ADPs (Figs. 5–7 and S4). The mechanism for selecting from the many chemically similar atoms needs to be investigated further. It appears that protein atoms form an intercalating mosaic of coexisting lattices (Fig. 6d), where it is common to see atoms from the same amino acid residues ending up in different clusters. Perhaps the most noticeable, but certainly not exclusive, example is the group of tryptophan residues, where the indole ring is often approximated as a rigid aromatic plane with torsional fluctuations around its two axes as the most important motions. Complicating this picture, the atoms within the indole ring cluster differently and share clusters with atoms in other tryptophan residues. Tryptophan residues are often difficult to replace by mutagenesis and play a key role in maintaining the thermodynamic stability of protein structures. They are one of the strongest promoters of ordered states^41,42^.

The disorder-to-order transition is also facilitated by asparagine and threonine residues^42^. In particular asparagine residues were poorly described by MD simulations, which predict larger B_eq_ and lower ANISO in absolute terms and relative to other amino acid types (Fig. 4C,B). It appears that in the folded form, the side chain amide of asparagine residues undergoes very limited fluctuations even in the absence of local interactions. Given the limited local contacts, MD simulations predict more positional freedom than is experimentally observed in our measurements. In the terahertz experiments, some of the asparagine atoms also appear to clusters together with catalytic residues (Fig. 6). A preliminary proposal is presented on how ADP similarity might be linked to structural constraints and to the symmetry between chemical groups in a protein in the Supporting Online Material, Figs. S4 and S5.

### Delocalized polar vibrations are important for structure and function prediction

Protein dynamics incorporates diffusive motions of folded and unfolded domains relative to one other (Fig. S6), conformational changes, elastic stretching (Fig. S6A), bending (Fig. S6C) and torsions. Rigid body approximation even at the level of small structural moieties such as aromatic side chains does not always provide natural clusters for ADP similarities. Our study demonstrates that within a folded unit polar terahertz vibrations can be identified (Fig. S6D). Importantly, the entrainment of these polar vibrations may have the ability to steer the orientation of involved atoms at great distances and the electron dynamics of covalent bonds provide additional local constraints for orienting the attached functional groups.

Physical proximity and local complementarity have been and still are the primary guiding principles in protein engineering. Local shape complementarity is necessary, but not sufficient condition for structure formation. Directed engineering of protein interfaces is less than straightforward due to the elusive (allosteric) influence of the rest of the structure. The similarity of parameters that describe fluctuations are not routinely analyzed. B_iso_-factors and NMR relaxation parameters by themselves do not contain much information: two atoms can easily have similar parameters, just by coincidence. Experimental and modeling errors make the comparison even more difficult. It requires series of observations^43,44^ in order to recognize the covariance of these parameters and assign multiple atoms to a specific functional role. With anisotropic ADPs, the chances of coincidental similarities are dramatically reduced. While multiple related structures help (as demonstrated in this study), it is possible to detect similarities between atoms with good confidence even in a single crystal structure. The examples described here show that the anisotropy of these fluctuations appears to have a functional relevance with two or more atoms with similar ADPs being responsible for a similar function (catalysis, conformational change between active and inactive form of the enzyme). These similarities can be revealed faster than time-consuming mutagenesis studies, which are also impossible when main chain atoms are concerned. While the extracellular enzyme trypsin is not typically seen as an allosteric enzyme, its substrate specificity, activation, and inhibitor binding involve diffusely distributed, concerted action of distant amino acid atom positions suggesting that the basis of allostery is a universal, emergent and dynamic process.

The identification of similar ADPs in a macromolecular structure is just the first essential step. The second and possibly more difficult step is to find and understand the geometric patterns between atoms belonging to the same ADP cluster. Since similar ADPs seem to be linked to the chemical nature of atoms, empirical geometric rules may be recognized or applied in the absence of ADP observations. These patterns would then assist the development of long-range ADP restraints, complementing restraints based on bonding connectivity and positional proximity. Improved ADP restraints may provide better electron density maps and more detailed structural models at lower crystallographic resolutions. More importantly, long-range geometric patterns between chemically similar atoms coupled to effective conformational sampling (simulated annealing) may be able to assist the *de novo* prediction of protein structures.

## Conclusions

Our study focused on two inseparable questions: the effect of terahertz radiation on protein crystals and the terahertz dynamics of folded proteins. We used crystallographic ADPs to measure the effect of terahertz radiation on crystals cooled to 100 K. Locally, we observed an increase in anisotropy and focused our attention on glycine and tryptophan residues, and the catalytic residue His-57, where the most obvious changes in anisotropy occur. The clustering of atoms based on their ADPs provided a relational map, which complemented the positional analysis of atoms based on their proximity. We performed NMA and MD simulations to predict ADPs and achieved only limited success. The clustering revealed that chemically similar atoms tend to have ADPs that are more similar. These atoms are distributed sparsely in the protein structure and belong to functionally connected amino acid residues.

## Methods

### Protein crystallization and flash cooling

Bovine trypsin (Sigma) was dissolved in 30 mM HEPES pH 7.0, 3 mM CaCl_2_ and 6 mg/ml benzamidine to obtain 60 mg/ml protein solution. Trypsin was crystallized using the hanging drop vapor diffusion method, by mixing 5 μl protein solution and 5 μl of precipitant solution (18% PEG8000, 50 mM HEPES pH 7.0, 0.2 M Ammonium sulfate, 3 mM CaCl_2_ and 6 mg/ml benzamidine). After 3 days, orthorhombic crystals appeared with approximate dimensions of 100–300 µm. The crystals were harvested and soaked in cryoprotectant solution (additional 33% glycerol) for 1–2 seconds prior to flash cooling in liquid nitrogen.

### X-ray diffraction data collection

X-ray diffraction data were collected at the EMBL P14 beamline in Hamburg. (Fig. S7) The incident X-ray beam was focused to a 10 µm horizontal × 50 µm vertical rectangular area at the sample position. The photon energy of the beam was 14 keV and the beam was attenuated to a photon flux of 4 × 10^11^ photons/s. Optimization of the experimental geometry allowed us to collect diffraction to higher resolution (wider angles) than previously^18^. The central panels of the Pilatus 6M-F detector were used (2463 × 2527 pixels) and the detector was placed at the distance of 130.4 mm from the crystal. We discuss the effect of X-ray radiation damage on ADPs in the Supporting Online Material and Fig. S8.

The crystals were oriented with the minikappa goniometer such that the normal vector of a clean crystal surface was aligned with the spindle direction. The beam cross section was positioned less than 100 μm from the crystal surface and 20 μm offset from the spindle axis. During X-ray exposure, the crystals were continuously rotated with 0.4 °/s while the Pilatus 6M-F was continuously recording images at 25 ms intervals. During each image recording period, the detector spent 22 ms with photon counting and 3 ms with I/O operations (read out).

The terahertz radiation was generated by an amplifier / frequency multiplier chain (X32 stage AMC, manufactured by Viginia Diodes Inc., Charlottesville, VA), which was driven by a MG3692c microwave signal generator (Anritsu). The input microwave radiation was set to 15.625 GHz (10 mW), which resulted in 0.5 THz radiation (1 mW). The diagonal horn antenna (WR-2.2) was positioned approximately at 0.5 cm from the crystals. Under the assumption of a Gaussian beam profile, the THz spot size was approximately 4 mm. This estimate assumes a waist radius of 1.3 mm and that the beam originates from approximately 1/3 inside the antenna. We examine the thermal effect of the terahertz radiation in the Supporting Online Material.

To reduce systematic differences associated with thermal effects, the terahertz radiation source was operated alternately. A DG645 pulse (delay) generator (Stanford Research Systems) was triggered on the raising edge of the EN OUT signal of the Pilatus 6M-F detector. Upon triggering, the pulse generator was programmed to send a 23.5 ms pulse to the terahertz radiation source (THz_on_ period). Simultaneously, the pulse generator was set to ignore subsequent triggers for 40 ms. Consequently, every odd numbered image was taken from the crystal in the terahertz irradiated state whereas the even numbered images recorded the diffraction image of the crystal in the non-irradiated state.

Odd and even numbered diffraction images still contained sufficiently super sampled measurements to integrate their total intensity independently^18,45^. This applies even for the widest-angle reflections recorded for this study, observed far away from the spindle axis.

### X-ray diffraction data analysis

The first diffraction image was always affected by the slow opening of the millisecond shutter and this image had systematically lower recorded scattering intensity. The data processing program XDS^46^ uses the first image of each data set for initial scale, which introduced a systematic difference between the data sets based on odd and even images. Therefore, the first odd and first even images of each data set were removed from subsequent analysis. In addition, the kappa goniometer shadow was identified in each data set and the affected images were removed. An even number of images were removed in order to maintain the equal number of odd and even numbered diffraction images.

The sets of diffraction images from the odd and even frames of each crystal were processed separately. By comparing the data and refinement statistics, we selected the crystals with generally high data quality, shown in Table S1. This pre-selection yielded 18 data sets, recorded from four reference and five terahertz radiated crystals. Moreover, since the frames affected by the goniometer shadow differed from crystal to crystal, the unaffected rotation wedges were integrated individually. The indexing and integration steps were carried out with the default settings in XDS. The subsequent scaling and merging were performed using the programs XSCALE and XDSCONV of the XDS package, respectively. In both programs, the standard settings for non-anomalous diffraction data were used.

### Model building and structural refinement

The subsequent model building and structural refinement were performed on each individual state (the odd and the even frames) of the individual crystals, using Refmac5 of the CCP4 package^47^. A single starting model was refined against the 18 data sets (9 crystals, recorded on odd and even diffraction images). Refinement statistics is shown in Table S1. This initial structure was based on pdb entry 4I8G, and the structure was solved by molecular replacement^48^. The starting model included 2067 non-hydrogen atoms, 288 of which belonged to water molecules, 20 were heteroatoms and 1759 were part of amino acid residues. Riding hydrogen atoms were included during refinement.

The individual crystal refinement was carried out with Refmac5, in three steps: rigid body, restrained isotropic and restrained anisotropic refinement procedures. Each refinement procedure was performed with the default settings for 100 cycles with an automatic weighting of restraints.

### ADP analysis and clustering

The diagonalization of the ADP tensor is defined as:

1 $${{\boldsymbol{U}}}_{{\boldsymbol{i}}{\boldsymbol{j}}}=[\begin{array}{ccc}{U}_{11} & {U}_{12} & {U}_{13}\\ {U}_{12} & {U}_{22} & {U}_{23}\\ {U}_{13} & {U}_{23} & {U}_{33}\end{array}]={\boldsymbol{P}}[\begin{array}{ccc}{E}_{1} & 0 & 0\\ 0 & {E}_{2} & 0\\ 0 & 0 & {E}_{3}\end{array}]{{\boldsymbol{P}}}^{-1}$$

where **U**_**ij**_ is the ADP tensor, **P** is the eigenvector matrix from the ADP tensor and E_1_, E_2_, E_3_ are eigenvalues of the ADP tensor. The eigenvalues and eigenvector are obtained by transforming the ADP tensor to its row echelon form: i.e by solving the equation ***Ux*** = ***0***, where ***x*** is a vector in R^3^ and ***0*** is the zero vector. Each atom in each crystal is described with two ***U***_***ij***_ matrices: in the “odd” and “even” state of the crystals. Odd and even states correspond to a “THz on” and a “THz off” state for THz irradiated crystals, respectively.

The equivalent B factors (B_eq_) are defined as:

2 $${B}_{eq}=8{\pi }^{2}\frac{1}{3}tr({{\boldsymbol{U}}}_{{\boldsymbol{ij}}})$$

where *tr(****U***_***ij***_) is defined as the trace of the ADP tensor (i.e. U_11_ + U_22_ + U_33_). The anisotropy value (ANISO) is defined as:

3 $$ANISO=\frac{{\rm{\min }}({E}_{1},{E}_{2},{E}_{3})}{{\rm{\max }}({E}_{1},{E}_{2},{E}_{3})}$$

i.e. the ratio of the minimum and maximum eigenvalue.

Through hierarchical clustering, all the observed atoms were iteratively grouped using the distance between their unmodified **U**_**ij**_ matrices. By having two observations of each atom in the same crystal, we obtained two symmetric **U**_**ij**_ matrices with six unique elements each.

We defined the Euclidian distance between atoms *u* and *v* using their experimental ***U***_***ij***_ matrices:

4 $$d(u,v)=\sqrt{\begin{array}{l}{({U}_{11,odd,u}-{U}_{11,odd,v})}^{2}+{({U}_{22,odd,u}-{U}_{22,odd,v})}^{2}+{({U}_{33,odd,u}-{U}_{33,odd,v})}^{2}+\\ {({U}_{12,odd,u}-{U}_{12,odd,v})}^{2}+{({U}_{13,odd,u}-{U}_{13,odd,v})}^{2}+{({U}_{23,odd,u}-{U}_{23,odd,v})}^{2}+\\ \,{({U}_{11,even,u}-{U}_{11,even,v})}^{2}+{({U}_{22,even,u}-{U}_{22,even,v})}^{2}+{({U}_{33,even,u}-{U}_{33,even,v})}^{2}+\\ {({U}_{12,even,u}-{U}_{12,even,v})}^{2}+{({U}_{13,even,u}-{U}_{13,even,v})}^{2}+{({U}_{23,even,u}-{U}_{23,even,v})}^{2}\end{array}}$$

The clustering was thus performed in this twelve-dimensional feature space. For clustering, Ward’s method was used^49^. Ward’s method groups the input data into individual clusters and selectively reorganize the clusters to minimize the total within-cluster variance. A smaller within-cluster variance groups the clusters into a closer branch. The calculations and visualization were performed with the help of the python libraries numpy, scipy, pandas^50^, cctbx^51^, seaborn and NGLView^52^.

### Apparent X-ray dose calculations

In order to compare the apparent absorbed doses in the different crystal structures first we estimated the cumulative absorbed X-ray dose corresponding to each diffraction image. The absorbed dose was calculated using the program RADDOSE 3D^53^. The apparent dose was calculated as the product of the dose per frame, multiplied with the weighted arithmetic mean of the number of frames for each crystal.

### Normal mode analysis and molecular dynamics simulations

Normal mode analysis (NMA) was performed using the Gromacs simulation package^54–56^ version 2018–2, compiled with double precision. The structure was first energy minimized rigorously by employing several cycles of Steepest Descents and Conjugate Gradients energy minimization cycles. The CHARMM36^57–59^ force field was used in all calculations. Electrostatics and van der Waals forces were truncated at 1.8 nm with a shift cutoff from 1.5 nm. After energy minimization, the Hessian matrix was diagonalized and all eigenvalues and eigenvectors were used except the first six rotational and translational modes. An ensemble of structures was generated at 100 K by the Gromacs program gmx nmens and ADPs were calculated by the Gromacs program gmx rmsf for all non-hydrogen protein atoms.

Molecular dynamics simulations were carried out using energies and forces as implemented in the OPLS-AA/L force field^60^. The protein was solvated in a cubic box so that the minimum distance between any protein atom and the edge of the box was 1.0 nm. The water molecules were approximated with the TIP3P model^61^. Cl^-^ ions were added in order to neutralize the charge. Before the MD simulation, internal constraints were relaxed by energy minimization. After the minimization, a restrained MD run was performed for 20 ps. During the restrained simulations, protein heavy atoms were fixed to their initial positions with a force constant of 1000 kJ mol^−1^ nm^−2^. The restraints were released and the system was equilibrated for 60 ns before data collection for analysis.

The temperature was kept constant (T = 300 K) by use of the velocity‐rescaling algorithm (τ_T_ = 0.1 ps)^62^. The pressure was coupled to an external bath with Berendsen’s coupling algorithm^63^ (P_ref_ = 1 bar, τ_P_=1 ps) during the equilibration and with the Parrinello–Rahman algorithm^64^ afterwards. Van der Waals forces were truncated at 1.0 nm with a plain cutoff. Long‐range electrostatic forces were treated using the particle mesh Ewald method^65^. Dispersion correction was applied for the energy and pressure.

After equilibration, the remaining 40 ns of simulations were divided into 10 ns segments and the ADPs were calculated with the Gromacs program gmx rmsf from the trajectory segments.

### Accession code

Crystal structures and X-ray diffraction data is available at the Protein Data Bank under entries: THz irradiated crystals (odd frames/even frames): 6SV0/6SUX, 6SV8/6SV6, 6SVB/6SV9, 6SVG/6SVD, 6SVJ/6SVI, Reference crystals (odd frames/even frames): 6SVR/6SVN, 6SVV/6SVU, 6SVX/6SVW, 6SW0/6SVZ.

## Supplementary information

Supplementary information
